# Comparative Study of Safety and Outcomes of Malecot and Pigtail Catheters in Ultrasound-Guided Percutaneous Nephrostomy: A Single-Center Retrospective Analysis

**DOI:** 10.7759/cureus.102732

**Published:** 2026-01-31

**Authors:** Gokul Kannan, Sajad A Para, Aamir B Raina, Abdul R Khawaja, Arif H Bhat, Sajad A Malik, Saqib Mehdi, Uvaisullah Quadir, Syed Shakeeb Arsalan

**Affiliations:** 1 Department of Urology, Sher-i-Kashmir Institute of Medical Sciences, Srinagar, IND

**Keywords:** catheter dislodgement, malecot catheter, percutaneous nephrostomy, pigtail catheter, ultrasound-guided nephrostomy, urinary tract obstruction

## Abstract

Purpose: Percutaneous nephrostomy (PCN) is a vital urological intervention to relieve urinary tract obstructions caused by stones, malignancies, or strictures, which can compromise renal function. PCN also facilitates urinary diversion in cases of leaks or fistulas. Catheter choice, such as Malecot (silicone) or pigtail (polyurethane), impacts outcomes, with complications like dislodgement and obstruction. This study compares the safety, efficacy, and patient outcomes of Malecot versus pigtail catheters in ultrasound-guided PCN.

Materials and methods: A retrospective observational comparative analysis was conducted at the Department of Urology, Sher-i-Kashmir Institute of Medical Sciences, Srinagar, India, from December 2022 to May 2025, involving 548 patients (248 Malecot, 300 pigtail) undergoing ultrasound-guided PCN for urinary obstruction due to urolithiasis, malignancy, or strictures. Demographic, procedural, and outcome data, including complication rates (per Clavien-Dindo classification), catheter displacement, and reintervention rates, were compared. Procedures utilized the Seldinger technique under ultrasound guidance. Statistical significance was assessed using p-values.

Results: Baseline demographics (age, sex, and hydronephrosis grade) were comparable, though the pigtail group had a higher Charlson Comorbidity Index (p=0.038). Malecot catheters showed superior performance in draining pus-laden obstructions (114/248 vs. 88/300, p<0.001), attributed to larger diameters (10f predominant, p<0.001). Pigtail catheters had higher displacement rates (15% vs. 6%, p<0.01) and reintervention rates (59% vs. 26%, p<0.001). Complication rates were similar (p=0.076), predominantly low-grade. Procedure times and hospital stays were equivalent.

Conclusion: In ultrasound-guided PCN, Malecot catheters were associated with lower displacement and reintervention rates than pigtail catheters in managing complex urinary obstructions, particularly pyonephrosis. These findings suggest a potential role for Malecot catheters in selected high-risk cases, though larger, multicenter studies are needed to refine catheter selection protocols.

## Introduction

Percutaneous nephrostomy (PCN) is a procedure commonly performed by urologists to relieve urinary obstruction and provide effective decompression of the collecting system. Obstruction of urinary outflow leads to elevated intrapelvic pressure, potentially causing nephron loss and renal atrophy if not addressed promptly. Common indications for PCN include obstructive urolithiasis and malignancies, requiring urgent intervention to safeguard kidney function and prevent sepsis [1]. In addition to relieving obstruction, PCN serves as an important modality for urinary diversion in conditions such as leaks, fistulas, or hemorrhagic cystitis, redirecting urine to bypass inflamed areas [2]. The procedure involves image-guided placement of a drainage catheter into the renal pelvis, commonly using ultrasound or fluoroscopy, to facilitate effective urine drainage. To maintain functionality and prevent issues like pyelonephritis, nephrostomy catheters are routinely replaced. Replacement intervals vary, typically ranging from six weeks to three months. Despite its widespread use, PCN is associated with procedure-related complications like bleeding, sepsis, or urine leakage, and catheter-related problems such as blockages or dislodgment. Among these, catheter dislodgment is a prevalent issue, with studies reporting incidence rates between 1% and 37.6%, often necessitating repeat intervention and increasing patient morbidity and healthcare costs [3,4].

Several factors influence catheter-related outcomes following PCN, such as tract length, catheter size, material composition, and design. While smaller-caliber catheters may improve patient comfort, larger-bore catheters and those with wider drainage windows offer superior performance in draining purulent or viscous contents by reducing clogging risk and improving flow dynamics [5]. Commonly used nephrostomy catheters, including Malecot catheters (made of silicone with winged retention tips) and pigtail catheters (made of polyurethane with curled retention tips), represent the two most commonly used catheter types in urological practice. Each has distinct advantages: pigtails are easier to place, while Malecot catheters may provide more stable anchoring and better drainage of thick infected material [5,6]. Despite ultrasound-guided PCN being a standard procedure for urologists, the choice between these catheters, Malecot and pigtail, remains debated due to varying material properties affecting outcomes like drainage efficiency and longevity. However, direct head-to-head comparisons, particularly under ultrasound guidance, are scarce. In routine clinical practice, catheter selection is often guided by patient-specific and intraoperative factors rather than standardized protocols. Despite the widespread use of both Malecot and pigtail catheters in ultrasound-guided PCN, no study has directly compared these catheter types in this specific clinical setting. The primary objective of this study was to compare catheter displacement and reintervention rates between Malecot and pigtail catheters in urologist-performed, ultrasound-guided PCN to provide real-world evidence to support clinical decision-making. This study also evaluates and compares the demographic and clinical characteristics and complication rates in a large single-center cohort.

## Materials and methods

Study design

This was a retrospective observational comparative study conducted at the Department of Urology, Sher-i-Kashmir Institute of Medical Sciences (SKIMS), Srinagar, Jammu and Kashmir, India, a tertiary care center, from December 2022 to May 2025. A total of 548 consecutive patients who underwent ultrasound-guided PCN were included and categorized into two groups based on the type of catheter used: Malecot (n=248) and pigtail (n=300) catheter groups.

A formal sample size calculation was not performed for this study. The sample size was determined by the number of eligible patients who underwent the procedure during the study period. As a result, the study may have limited statistical power to detect small or modest differences between groups, particularly for secondary outcomes. Therefore, nonsignificant findings should be interpreted with caution, as the absence of statistical significance does not necessarily exclude clinically meaningful differences.

Ethical considerations

The study was approved by the Institutional Ethics Committee (IEC), Sher-i-Kashmir Institute of Medical Sciences (SKIMS), Srinagar. This study was conducted in accordance with the ethical principles outlined in the Declaration of Helsinki (2013). Given the retrospective observational design, the requirement for written informed consent for data analysis was waived by the Institutional Ethics Committee. Informed consent was obtained from all patients for the PCN procedure as per institutional protocol.

Inclusion criteria 

Inclusion criteria included patients with urinary tract obstruction secondary to urolithiasis, malignancy, or strictures requiring PCN; ultrasound-guided PCN performed by urologists using either Malecot or pigtail catheters at the study center during the study period; and the availability of complete procedural and outcome data.

Exclusion criteria

Exclusion criteria included PCN procedures performed under fluoroscopic guidance, PCN performed by non-urologists, the use of alternative catheter types other than Malecot or pigtail (e.g., balloon or locking-loop catheters), and refusal of informed consent for the procedure or for the use of clinical data, where applicable.

Catheter selection criteria

Catheter type (Malecot or pigtail) was selected at the discretion of the operating urologist based on clinical presentation, imaging findings, and anticipated drainage requirements. Malecot catheters were preferentially used in complex or infected obstructive conditions, including pyonephrosis or thick purulent drainage, where larger lumen drainage was considered advantageous. Pigtail catheters were more commonly utilized in uncomplicated obstructions requiring temporary decompression. Given the absence of established comparative evidence guiding catheter choice in ultrasound-guided PCN, no standardized allocation protocol or randomization was employed. As this was a retrospective, non-randomized study reflecting real-world clinical practice, no standardized allocation protocol was employed. The authors acknowledge that this approach may introduce confounding by indication. To address this, prespecified subgroup analyses were planned based on catheter diameter, presence of pus at initial drainage, Charlson Comorbidity Index (CCI) category, and laterality (unilateral vs. bilateral PCN). These analyses were performed to explore the potential influence of baseline clinical differences on outcomes.

Technique

All procedures were performed by trained urologists. Patients were positioned in the prone position based on anatomical accessibility and comfort. Local anesthesia (2% lidocaine, 10-20 mL) was administered at the puncture site. A high-frequency ultrasound probe (5-10 MHz) was used to visualize the renal pelvis. 

**Figure 1:**
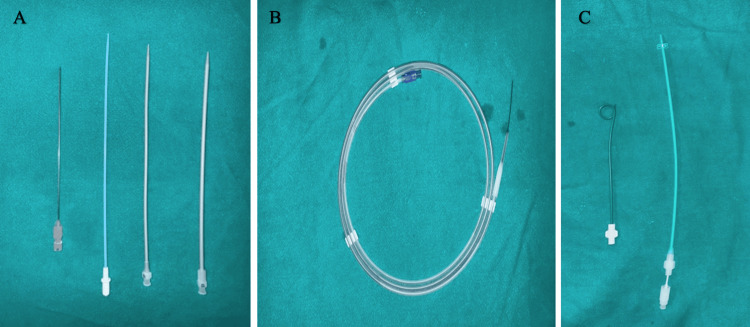
Instruments used for the procedure: (A) 18G, 15 cm puncture needle and 8f, 10f, and 12f Teflon dilators; (B) 0.032 PTFE straight-tip guide wire; and (C) pigtail and Malecot catheters PTFE, polytetrafluoroethylene

**Figure 2:**
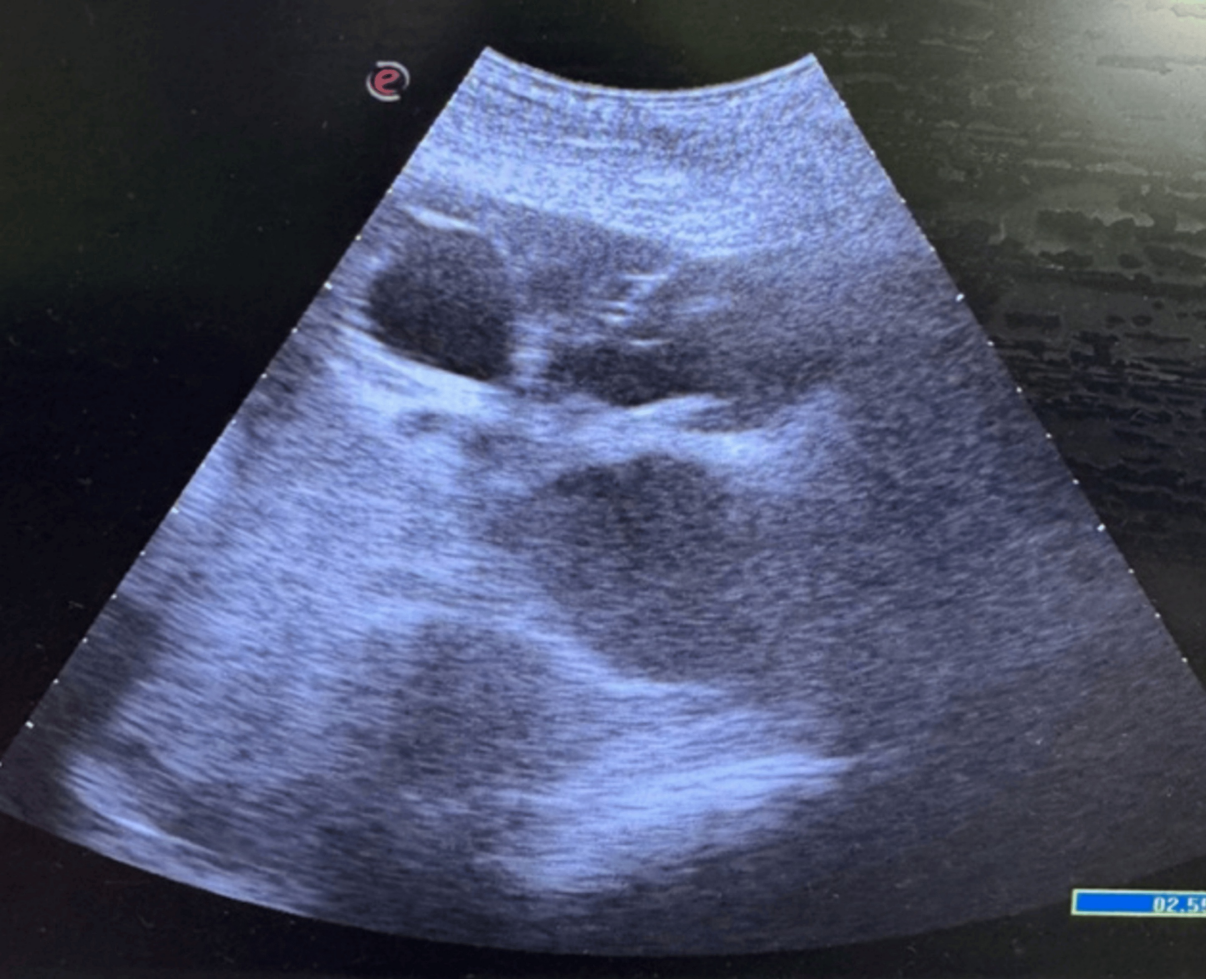
Ultrasound-guided puncture of a dilated pelvicalyceal system

PCN was performed using the Seldinger technique. An initial puncture of the desired dilated calyx was done by an 18G puncture needle (Figure 1) under ultrasound guidance (Figure 2), followed by the insertion of a 0.032 polytetrafluoroethylene (PTFE) straight-tip guide wire. The tract was dilated serially by 8f, 10f, and 12f Teflon dilators. An appropriately sized pigtail catheter (8f or 10f, 22 cm polyurethane-based with a curled tip) or Malecot catheter (silicone-based with winged retention) was introduced over the guide wire and secured. Following the placement of the nephrostomy catheter into the pelvis, all catheters were secured to the skin at the puncture site with multiple stitches and knots to avoid dislodgement. Prophylactic antibiotics (e.g., ceftriaxone 1 g IV) were administered to reduce infection risk, with post-procedure care including regular catheter flushing and site monitoring. Additionally, all patients and companions were instructed clearly and in detail about catheter care and what needs to be done to prevent catheter dislodgement. Despite all the precautions outlined, spontaneous nephrostomy catheter dislodgements continue to be among the most significant catheter-related complications.

Outcome Definitions

Catheter displacement was defined as partial or complete migration of the nephrostomy catheter from its intended intrarenal position requiring repositioning or replacement. Catheter patency duration was defined as the time interval from catheter insertion to documented catheter dysfunction due to blockage, displacement, or elective removal. Reintervention was defined as any unplanned procedure required to restore urinary drainage, including catheter replacement, upsizing, or repeat PCN.

Procedural and Post-procedural Protocols

All PCN procedures were performed under ultrasound guidance using a standardized Seldinger technique by experienced urologists. Catheter type and size were selected based on clinical judgment, as described previously.

Post-procedural care followed a standardized institutional protocol, including catheter fixation, routine catheter care, monitoring of urine output, and clinical assessment for complications. Imaging was performed when clinically indicated.

Catheter displacement was confirmed based on clinical assessment and/or imaging findings documented in the medical record. Reintervention events were identified through procedure records and operative notes. Outcome assessment was performed by the treating clinical team as part of routine patient care. Follow-up was conducted during the index hospitalization and subsequent outpatient visits as per standard institutional practice. Patients were followed until catheter removal or the study endpoint, and complications were recorded according to the Clavien-Dindo classification.

Methodology

To evaluate the safety and efficacy of both tubes, several parameters were analyzed: (1) patient demographics, including age, sex, comorbidities (CCI), side (unilateral/bilateral), grade of hydronephrosis (HDN), and indications for PCN; (2) intraprocedural parameters, including timing from diagnosis to intervention, procedure time, presence of pus, hospital stay or day care, type of tube, and catheter diameter; (3) post-procedure complications, including bleeding, sepsis, hematuria, retroperitoneal hematoma, extravasation and urinoma, stuck guidewire, perforation of adjacent viscera such as the colon, hydrothorax and pneumothorax, surgical site infection (SSI), blockage of the catheter, catheter dislodgement, retained tip of the catheter, arteriovenous (AV) fistula, pseudoaneurysm, and ureteric stricture, all graded according to the Clavien-Dindo classification; and (4) catheter patency duration and reintervention rates.

Statistical Analysis

Data were collected and entered into Microsoft Excel (Redmond, WA, USA) and subsequently analyzed using IBM SPSS Statistics version 25 (IBM Corp., Armonk, NY). Continuous variables were expressed as mean±standard deviation or median with interquartile range, depending on data distribution, and were compared between groups using the Student’s t-test or the Mann-Whitney U test, as appropriate. Categorical variables were summarized as frequencies and percentages and compared using the chi-square test or Fisher’s exact test. Missing data were minimal and were handled using complete-case analysis without imputation. A two-sided p-value ≤0.05 was considered statistically significant. Given the retrospective, nonrandomized design of the study, no multivariable adjustment was performed for baseline imbalances; therefore, the results should be interpreted with caution.

## Results

In our study, a total of 548 patients underwent ultrasound-guided PCN, of whom 300 received pigtail catheters and 248 received Malecot catheters. Baseline demographic and clinical characteristics of the two groups are summarized in Table 1. There were no statistically significant differences between the groups with respect to age distribution (chi-square test, p=0.251) or sex (chi-square test, p=0.516). The pigtail group comprised 8.7% (95% CI 6.0%-12.4%) patients aged <20 years, 25.7% (95% CI 21.1%-30.9%) aged 20-40 years, 35.0% (95% CI 29.8%-40.6%) aged 40-60 years, and 30.7% (95% CI 25.7%-36.1%) aged >60 years, with 57.3% male (95% CI 51.7%-62.8%) and 42.7% female (95% CI 37.2%-48.3%). The Malecot group had 4.4% (95% CI 2.5%-7.8%) aged <20 years, 27.4% (95% CI 22.2%-33.3%) aged 20-40 years, 34.7% (95% CI 29.0%-40.8%) aged 40-60 years, and 33.5% (95% CI 27.9%-39.6%) aged >60 years, with 60.1% male and 39.9% female. CCI severity differed significantly between the two groups (p=0.038). No statistically significant differences were found in laterality (chi-square test, p=0.297) or HDN grade (chi-square test, p=0.140), with the pigtail group showing 61.0% unilateral (95% CI 55.4%-66.3%) and 39.0% bilateral (95% CI 33.7%-44.6%) PCNs, and the Malecot group showing 65.3% unilateral (95% CI 59.2%-71.0%) and 34.7% bilateral (95% CI 29.0%-40.8%). HDN grades in the pigtail group were 8.3% Grade 1 (95% CI 5.7%-12.0%), 27.3% Grade 2 (95% CI 22.6%-32.6%), 39.3% Grade 3 (95% CI 34.0%-45.0%), and 25.0% Grade 4 (95% CI 20.4%-30.2%), versus 4.8% Grade 1 (95% CI 2.8%-8.3%), 22.2% Grade 2 (95% CI 17.5%-27.8%), 43.5% Grade 3 (95% CI 37.5%-49.8%), and 29.4% Grade 4 (95% CI 24.1%-35.4%) in the Malecot group.

**Table 1:** Demographic and clinical characteristics CCI, Charlson Comorbidity Index; HDN, hydronephrosis

Parameter	Pigtail (n=300)	Malecot (n=248)	Remarks
Age			P-value=0.251, Chi-square value (X^2^)=4.10
<20 years	26	11	
20-40 years	77	68	
40-60 years	105	86	
>60 years	92	83	
Sex			P-value=0.516, Chi-square value (X^2^)=0.32
Male	172	149	
Female	128	99	
CCI			P-value=0.038
Mild	127	124	
Moderate	114	94	
Severe	59	30	
Side			P-value=0.297, Chi-square value (X^2^)=1.09
Unilateral (U/L)	183	162	
Bilateral (B/L)	117	86	
Grade of HDN			P-value=0.140, Chi-square value (X^2^)=5.46
Grade 1	25	12	
Grade 2	82	55	
Grade 3	118	108	
Grade 4	75	73	

Procedural and outcome characteristics are detailed in Table 2. Procedural indications for PCN were comparable (chi-square test, p=0.351) between the two groups. Timing from diagnosis to PCN placement (p=0.927) and median procedure time (15 minutes) were equivalent, with 63.3% of the pigtail group (95% CI 57.7%-68.6%) and 63.7% of the Malecot group (95% CI 57.6%-69.4%) placed within 24 hours. The proportion of patients managed as daycare cases versus those requiring hospital admission was comparable between the two groups (p=0.861). The presence of pus was significantly higher in the Malecot group compared to the pigtail group (46.0% vs. 29.3%, chi-square test, p<0.001). Catheter diameter differed significantly (chi-square test, p<0.001), with the pigtail catheters predominantly 8f and Malecot catheters predominantly 10f. Given the close association between catheter diameter, catheter design, and underlying disease severity, the observed differences in outcomes likely reflect a combined effect of catheter type and catheter diameter rather than catheter design alone. Clavien-Dindo complications showed no significant difference (chi-square test, p=0.076), with the majority of complications being Grades I-II in 94.0% of the pigtail group and 97.2% of the Malecot group, and severe complications (Grades III-V) in 6.0% and 2.8%, respectively.

**Table 2:** Procedural and outcome characteristics

Parameter	Pigtail (n=300)	Malecot (n=248)	Remarks
Indications for PCN			P-value=0.351, X^2^=5.56
Obstructive stone disease	88	67	
Malignancy	137	114	
Pyonephrosis	30	25	
Ureteric stricture	20	11	
Post-op urine leak	15	13	
Other causes	10	18	
Timing from diagnosis to PCN placement			P-value=0.927, X^2^=0.01
<24 hours	190	158	
>24 hours	110	90	
Procedure time	15 mins	15 mins	
Presence of pus			P-value=<0.001, X^2^=18.6
Yes	88	114	
No	212	134	
Length of hospital stay			P-value=0.861, X^2^=0.03
Hospital stay	145	118	
Day care	155	130	
Catheter diameter			P-value=<0.001, X^2^=165.3
8f	220	66	
10f	78	170	
12f	2	12	
Clavien-Dindo grading			P-value=0.076, X^2^=3.17
Grades I, II	282	241	
Grades IIIa, IIIb, IV, V	18	7	

**Figure 3:**
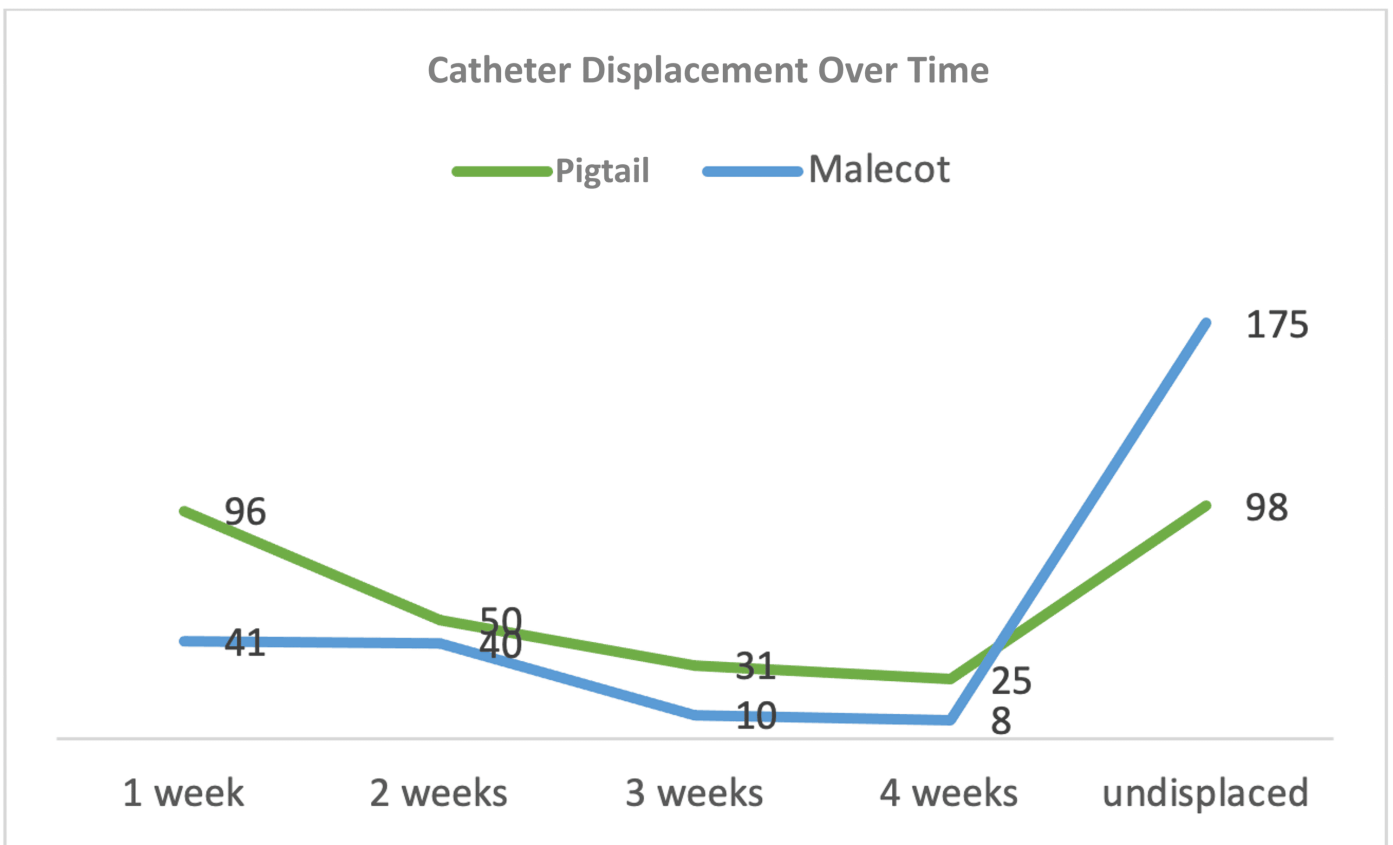
Catheter displacement over time This line chart visualizes catheter displacement rates over time (one to four weeks) for both groups.

Catheter displacement was observed more frequently in patients managed with pigtail catheters (Table 3 and Figure 3) (67.3%, 95% CI 61.9%-72.4% vs. 29.4%, 95% CI 24.1%-35.4%; OR 4.96, 95% CI 3.45-7.14), with events within one week at 32.0% for the pigtail group vs. 16.5% for the Malecot group, within two weeks at 16.7% vs. 5.6%, within three weeks at 10.3% vs. 4.0%, and within four weeks at 8.3% vs. 3.2%. The reintervention rate was significantly higher in the pigtail group (59.0%, 177/300, 95% CI 53.4%-64.4% vs. 26.2%, 65/248, 95% CI 21.1%-32.0%; OR 4.05, 95% CI 2.81-5.83). Reinterventions were further subclassified based on the primary indication for repeat intervention. These included catheter dislodgement, catheter blockage or poor drainage, planned upsizing, and disease progression requiring repeat decompression. The majority of reinterventions in both groups were related to mechanical factors, predominantly catheter dislodgement, followed by functional causes such as catheter blockage. Planned upsizing and disease progression accounted for a smaller proportion of reinterventions. On nonparametric analysis, the overall burden of catheter displacement and reintervention differed significantly between the two groups (Mann-Whitney U=27,218; Z=-6.94; p<0.001).

**Table 3:** Catheter displacement and reintervention rate

Parameter	Pigtail (n=300)	Malecot (n=248)
Catheter displacement		
Within 1 week	96	41
Within 2 weeks	50	14
Within 3 weeks	31	10
Within 4 weeks	25	8
Undisplaced	98	175
Reintervention rate	177/300	65/248

While formal catheter patency duration was not quantified as a continuous time-to-event variable, time to catheter displacement was analyzed as a clinically meaningful surrogate for catheter durability. This approach was adopted given the retrospective nature of follow-up and variability in the timing of elective catheter removal or exchange. 

Stratified analysis

To address baseline imbalances and potential confounding by indication, subgroup analyses were performed based on clinically relevant variables. Among patients with purulent drainage at initial PCN placement, catheter displacement was observed in 49 of 88 patients (55.7%) managed with pigtail catheters compared to 23 of 114 patients (20.2%) managed with Malecot catheters, demonstrating a higher rate of catheter displacement in the pigtail group within this high-risk subgroup. Similar directional trends in catheter displacement and reintervention were observed when analyses were stratified by catheter diameter (8f vs. ≥10f), CCI category, and laterality, without material alteration of the findings (Table 4).

**Table 4:** Stratified analysis of catheter displacement according to the presence of purulent drainage

Subgroup	Pigtail	Malecot
Presence of pus - yes	49/88 (55.7%)	23/114 (20.2%)

## Discussion

This study evaluates the performance of pigtail and Malecot catheters in ultrasound-guided PCN for managing urinary obstruction, focusing on demographic profiles, procedural characteristics, safety, and critical clinical outcomes. Our findings demonstrate that Malecot catheters were associated with improved drainage in infected systems and lower catheter displacement and reintervention rates, despite comparable baseline demographics and procedural metrics. These results align with and extend existing literature on PCN catheter performance, highlighting the impact of catheter design on drainage efficiency and retention stability, particularly in complex cases involving viscous or infected effusions.

Demographic and clinical characteristics

This comparative study of ultrasound-guided PCN with pigtail (n=300) versus Malecot (n=248) catheters revealed comparable baseline characteristics in age (p=0.251), sex (p=0.516), laterality (p=0.297), HDN grade (p=0.140), indications (p=0.351), and time from diagnosis to intervention (p=0.927), minimizing confounding from these factors. However, a statistically significant difference in CCI (p=0.038) indicated a higher burden of moderate (114/300 vs. 94/248) and severe (59/300 vs. 30/248) comorbidities in the pigtail group, potentially influencing catheter stability in frailer patients.

Procedural and outcome characteristics

There was a significant difference in the presence of pus at the time of nephrostomy placement (p<0.001), with the Malecot group demonstrating a higher proportion of infected systems (114/248 vs. 88/300, p<0.001), reflecting selective use in clinically suspected pyonephrosis, as well as their larger diameter and wider drainage windows. This disparity likely stems from the pigtail’s narrower side holes and coiled configuration, which are prone to clogging by thick purulent material in pyonephrotic kidneys. Thus, the Malecot cohort underscores its suitability for pyonephrosis, potentially decreasing antibiotic dependence and sepsis risk, thus supporting its preferential use in suspected infected obstructions. Larger-diameter catheters with wider drainage windows have been shown to improve patency and reduce clogging in viscous or infected systems, supporting the preferential use of larger-bore catheters in such situations, as stated by Iyer D et al. [5].

Procedure times (15 minutes) and hospital stay patterns (p=0.861) were comparable, reflecting the efficiency of ultrasound-guided PCN irrespective of catheter type. There was a significant difference in catheter diameter distribution (p<0.001). This reflects clinical selection favoring larger Malecot diameters for anticipated high-viscosity drainage, consistent with PCN practice guidelines. Catheter diameter represents an important confounding factor when comparing nephrostomy catheter outcomes. In the present study, Malecot catheters were more commonly used in larger diameters, while pigtail catheters were predominantly smaller. As catheter diameter itself may influence drainage efficiency, patency, and risk of displacement, the observed differences in outcomes should be interpreted as reflecting a combined effect of catheter design, catheter diameter, and underlying clinical severity rather than catheter design alone. This limitation is inherent to the retrospective, real-world nature of the study. Catheter material also plays an important role in drainage efficiency, with silicone-based catheters demonstrating better resistance to encrustation and biofilm formation than polyurethane catheters, as highlighted by Iyer D et al. [5]. Recent evidence has also shown that silicone-based urinary drainage catheters are associated with a lower risk of bacterial colonization and catheter-related infection compared to polyurethane catheters, particularly during prolonged indwelling periods, although these outcomes remain influenced by catheter dwell time, design characteristics, and patient-related factors, as reported by Waqas et al. [7].

Complication rates, assessed via Clavien-Dindo grading, showed no significant difference (p=0.076), with most events low-grade. This finding is consistent with contemporary studies demonstrating that ultrasound-guided PCN is a safe procedure with low rates of severe complications when performed by trained urologists, with reported technical success rates exceeding 95%, as noted by Wang et al. [8].

Catheter displacement and reintervention

Figure 3 illustrates catheter displacement rates over one to four weeks, with pigtail catheters showing higher cumulative displacement (15% vs. 6% for Malecot; p<0.01), peaking at week two in both groups but resolving faster in the Malecot arm. Dislodgement of nephrostomy tubes is a well-recognized problem that often necessitates repeat intervention and increases patient morbidity, as noted by Radecka et al. [9]. In selected cases, salvage of dislodged nephrostomy catheters through the original tract may be feasible, although technical failure can occur depending on tract maturity and catheter-related factors, according to Ozawa et al. [10]. Catheter design is an important determinant of displacement risk, with curled pigtail catheters being more prone to migration compared to catheters with broader anchoring mechanisms, as shown by Panach-Navarrete et al. [4] and Niwa et al. [11]. The lower displacement rates observed with Malecot catheters in this study may be attributed to their winged retention design, which provides improved resistance to traction forces and patient movement. Reduced displacement translated into a significantly lower reintervention rate in the Malecot group, highlighting catheter stability as a critical determinant of long-term success following PCN. These findings are consistent with outcome-focused series demonstrating that improved procedural success and patient outcomes following PCN are influenced by complication burden and clinical context, as reported by Parmar et al. [12].

Table 3 quantifies displacement-related reinterventions, with pigtail catheters incurring a 59% reintervention rate compared to 26% for Malecot catheters (p<0.001). This highlights the potential advantage of Malecot catheters in reducing procedural burden and improving patient outcomes. The predominance of mechanically driven reinterventions, particularly catheter dislodgement, suggests that differences between catheter types may be more closely related to anchoring characteristics rather than drainage capacity alone. Functional causes such as blockage were less frequent, supporting the interpretation that catheter stability plays a central role in determining the need for reintervention in ultrasound-guided PCN. This significant difference between the two catheter groups highlights reintervention as a clinically meaningful endpoint that reflects catheter stability, functional longevity, and adequacy of drainage.

The findings of this study should be interpreted as supportive evidence. Catheter selection in ultrasound-guided PCN should remain individualized, integrating patient characteristics, procedural context, and operator judgment within routine clinical practice. Baseline differences in comorbidity burden and catheter size were present between groups. Although stratified analyses were performed to mitigate their potential confounding effects, residual confounding cannot be excluded due to the retrospective nature of the study.

Limitations

This study is limited by its retrospective, nonrandomized design, which may have introduced selection bias, as catheter selection was based on clinical judgment rather than random allocation. Baseline differences in comorbidity burden and catheter diameter were present between groups; although stratified analyses were performed to mitigate potential confounding, residual confounding cannot be fully excluded. These imbalances reflect real-world, clinician-driven catheter selection but limit causal attribution to catheter design alone. Consequently, the observed associations should be interpreted as hypothesis-generating rather than definitive evidence of catheter superiority. The absence of a formal sample size calculation may limit statistical power for detecting smaller differences. Although catheter patency duration was predefined in the study methodology, quantitative reporting of median patency time or time-to-failure analysis was limited by the retrospective study design and heterogeneity in catheter removal practices. Consequently, time to catheter displacement and the need for reintervention were used as pragmatic surrogate markers of catheter durability, which are clinically relevant and commonly reported outcomes in PCN studies. This was a single-center study with follow-up limited to the early postprocedural period, which may affect the generalizability of the findings and preclude assessment of long-term outcomes.

## Conclusions

In this large retrospective comparative study of ultrasound-guided PCN, both pigtail and Malecot catheters were effective in achieving urinary diversion. However, Malecot catheters were associated with lower catheter displacement and reintervention rates compared with pigtail catheters. This difference was particularly evident in complex and infected obstructive scenarios, such as pyonephrosis, where effective drainage and catheter stability are critical to clinical outcomes. Importantly, overall complication rates, procedural duration, timing of intervention, and length of hospital stay were comparable between the two catheter types, with the majority of complications being low-grade. These findings suggest that catheter design and diameter play a meaningful role in maintaining patency and reducing downstream interventions following ultrasound-guided PCN, especially in high-risk clinical settings.

While the retrospective, nonrandomized nature and the presence of confounding by indication warrant cautious interpretation, the large sample size and real-world data provide valuable insights into catheter performance in routine urological practice. Taken together, the results indicate an association between Malecot catheter use and improved catheter stability in selected high-risk clinical settings, while acknowledging that catheter choice, size, and underlying disease severity are closely interrelated. Future prospective, multicenter studies are required to validate these findings and to develop evidence-based catheter selection algorithms.
